# Kallikrein–Kinin System Suppresses Type I Interferon Responses: A Novel Pathway of Interferon Regulation

**DOI:** 10.3389/fimmu.2018.00156

**Published:** 2018-02-02

**Authors:** Alecia Seliga, Michael Hweemoon Lee, Nicole C. Fernandes, Viviana Zuluaga-Ramirez, Marta Didukh, Yuri Persidsky, Raghava Potula, Stefania Gallucci, Uma Sriram

**Affiliations:** ^1^Department of Pathology and Laboratory Medicine, Temple University, Philadelphia, PA, United States; ^2^Laboratory of Dendritic Cell Biology, Department of Microbiology and Immunology, Lewis Katz School of Medicine, Temple University, Philadelphia, PA, United States

**Keywords:** kallikrein–kinin system, bradykinins, ACE inhibitors, dendritic cells, PBMC, TLR, Type I IFNs, lupus

## Abstract

The Kallikrein–Kinin System (KKS), comprised of kallikreins (klks), bradykinins (BKs) angiotensin-converting enzyme (ACE), and many other molecules, regulates a number of physiological processes, including inflammation, coagulation, angiogenesis, and control of blood pressure. In this report, we show that KKS regulates Type I IFN responses, thought to be important in lupus pathogenesis. We used CpG (TLR9 ligand), R848 (TLR7 ligand), or recombinant IFN-α to induce interferon-stimulated genes (ISGs) and proteins, and observed that this response was markedly diminished by BKs, klk1 (tissue kallikrein), or captopril (an ACE inhibitor). BKs significantly decreased the ISGs induced by TLRs *in vitro* and *in vivo* (in normal and lupus-prone mice), and in human PBMCs, especially the induction of *Irf7* gene (*p* < 0.05), the master regulator of Type I IFNs. ISGs induced by IFN-α were also suppressed by the KKS. MHC Class I upregulation, a classic response to Type I IFNs, was reduced by BKs in murine dendritic cells (DCs). BKs decreased phosphorylation of STAT2 molecules that mediate IFN signaling. Among the secreted pro-inflammatory cytokines/chemokines analyzed (IL-6, IL12p70, and CXCL10), the strongest suppressive effect was on CXCL10, a highly Type I IFN-dependent cytokine, upon CpG stimulation, both in normal and lupus-prone DCs. klks that break down into BKs, also suppressed CpG-induced ISGs in murine DCs. Captopril, a drug that inhibits ACE and increases BK, suppressed ISGs, both in mouse DCs and human PBMCs. The effects of BK were reversed with indomethacin (compound that inhibits production of PGE2), suggesting that BK suppression of IFN responses may be mediated *via* prostaglandins. These results highlight a novel regulatory mechanism in which members of the KKS control the Type I IFN response and suggest a role for modulators of IFNs in the pathogenesis of lupus and interferonopathies.

## Introduction

The kallikrein–kinin system (KKS) is a metabolic cascade that releases vasoactive kinins (bradykinin-related peptides) upon triggering and has many pharmacological activities; KKS is involved in inflammation and cooperates with important pathways like the renin–angiotensin, coagulation, and complement (1–3). Bradykinin (BK) is the end product of the KKS that is formed from plasma or tissue kallikreins (klks) (4) and acts *via* their receptors (B1 or B2) (5) to induce responses *via* nitric oxide and/or prostaglandin production (3). The angiotensin-converting enzyme (ACE), which is part of the renin-angiotensin system (6), not only releases angiotensin II but also inactivates BK (6–8). Inhibitors of ACE have been used successfully in a variety of clinical conditions related to heart or kidney functions (9). The beneficial effects of administration of ACE inhibitors (ACEis) have been attributed to enhancing the actions of BK *via* its receptor (1, 7).

There is increasing evidence that the KKS is involved in systemic lupus erythematosus (SLE) (10–12). Giving exogenous klks has been shown to ameliorate lupus in mice, and lupus-prone mice have been found to produce less klks than wild-type mice (12). SLE is a systemic autoimmune disease of complex etiology in which immune complex deposition and complement activation lead to inflammation and tissue damage (13). Type I IFN signature is the hallmark of the disease, and efforts have been underway to attenuate the IFN responses in lupus (14–18). Current therapeutic candidates under serious testing include agents that block Type I IFN directly (16) or IFN generated *via* TLR ligands as TLR7/9 (19, 20). No link has so far been made between Type I IFNs and the KKS.

Molecules of the KKS have been shown to ameliorate end-stage kidney disease in mouse models (klk and BK) (11, 21) and in lupus patients (ACEis) (10) So far, the use of BKs and ACEis have been focused mainly on the renal- and cardio-protective effects, especially treatment upon onset of glomerulonephritis (GN) (22). In this study, we asked the question if exogenous KKS decreases the IFN responses induced by either TLR (TLR7 and TLR9) or direct IFN-α stimulation in normal and lupus-prone mice, as well as in human PBMCs. We show that BKs and other molecules of the KKS system (klks and ACEi) can suppress an ongoing Type I IFN response by decreasing the interferon-stimulated gene (ISG) expression, especially IRF7 the master regulator of Type I IFNs, both in mouse and human cells. Our results strongly suggest a novel pathway of Type I IFN regulation by the KKS.

## Materials and Methods

### Mice

Female C57BL/6 (B6) mice and B6.NZM Sle1/Sle2/Sle3 (Sle) mice were bought from The Jackson Laboratory and used between 7 and 12 weeks of age. We have shown before that Sle mice are pre-disease at this age and do not make any detectable autoantibodies (23). We used only female mice because of the higher prevalence of Sle in women than men (9:1 ratio, women to men) (24). Animals were maintained in the animal facility in accordance to the guidelines of the Institutional Animal Care and Use Committees of Temple University, which is an American Association for the Accreditation of Laboratory Animal Care-accredited facility.

### Reagents

The following reagents were used to stimulate cells: Recombinant human IFN-α (1,000 U/ml, PBL Assay Science, Piscataway, NJ, USA), mouse IFN-α (1,000 U/ml, PBL Assay Science), CpG-B 1826 (10 µg/ml), CpG-A-2336 (5 µg/ml) (IDT Biotechnologies, Coralville, IA, USA), CpG-A-1585 (1 µg/ml) (Invivogen, San Diego, CA, USA), resiquimod (1 µg/ml) (R848; Invivogen) (23, 25–27), bradykinin peptide (10 µM), Lys-des-Arg(9)-bradykinin, which is a kinin breakdown product and a selective bradykinin B1 receptor agonist (10 µM), Arg–Pro–Hyp–Gly–Phe–Ser–Pro–Phe–Arg B2 receptor agonist (10 µM), B1 receptor antagonist ([des-Arg^10^-HOE140]- DH-1 μg/ml), B2 receptor antagonist (HOE140–H–10 μM) (all bradykinin agonists and antagonists were purchased from Sigma-Aldrich, St. Louis, MO, USA) (28), recombinant human klk1 (1 µg/ml) (Creative Biomart, Shirley, NY, USA), captopril (20 μM)(Sigma) (29), and indomethacin (indo) (1 µg/ml) (Sigma) (30).

### Murine Bone Marrow Dendritic Cell (DC) Culture

We used bone-marrow-derived dendritic cells (BMDCs) as our murine cell model. BMDCs were generated as previously described (27). Briefly, bone marrow precursors were flushed from femurs and tibias of mice and the cells were seeded at 1 million cells/well in complete IMDM (Mediatech, Manassas, VA, USA) (10% FBS, penicillin/streptomycin, gentamicin, and 2-ME) (Life Technologies, Grand Island, NY, USA) enriched with 3.3 ng/ml GMCSF (BD Biosciences, San Jose, CA, USA) in 24-well plates. One milliliter of medium was added on day 2, and half was replaced on day 5 and subsequently each day until the culture was used (day 6/7). Some experiments were also performed using bone marrow precursors that were differentiated into DCs in medium containing Flt3L (23, 27). Briefly, bone marrow cavities were flushed, and cells were plated at 1 million cells/well in a 24-well plate in complete RPMI medium containing 10% FBS, l-glutamine, penicillin/streptomycin, 2-mercaptoethanol, and 10% supernatant from Flt3L cell line as described in Ref. (27). Cells were stimulated and harvested on day 8 or 9. Resting DC cultures were stimulated and BMDCs were harvested after 7 h of stimulation for RNA analysis and after 24 h for FACS analysis of surface activation markers. Culture supernatants were collected at the same time-points, for cytokine analysis by ELISA.

### Isolation of Human PBMCs

PBMCs were isolated from leukotrap filters (31) from normal human donors (from Red Cross, Philadelphia, PA, USA). The samples received from American Red Cross, used in this study, were de-identified samples and no information about the donors is available to the investigators. Briefly, leukotrap filters were flushed with PBS containing 5 mM EDTA and spun at 400 *g* at room temperature (RT) without brakes for 10 min. The clear supernatant was removed, and the blood was layered onto Ficoll–Hypaque (GE Healthcare Lifesciences, Pittsburg, PA, USA) and spun at RT for 40 min at 400 *g* without brakes. The buffy interface was isolated, washed in medium, counted and plated at 2.5 million cells/ml in 24-well plates in complete RPMI medium containing 10% FBS, penicillin/streptomycin and 10 mM HEPES.

### *In Vivo* Injections

B6 or Sle mice were injected intraperitoneally with CpG-A-1585 (10 μg/mouse) overnight to induce IFN responses. BK (100 µM) was injected *via* retro-orbital route and after 2 h mice were euthanized, and spleens were harvested for gene expression analysis.

### Magnetic Bead Sorting of Splenic DCs and *Ex Vivo* Stimulation

Dendritic cells from B6 mice were sorted using MACS beads (Miltenyi Biotech, Auburn, CA, USA), stimulated and harvested for gene expression analysis. Briefly, spleens were incubated in medium containing 8 mg/ml collagenase (Worthington Biochemical Corporation, Lakewood, NJ, USA) and 1,000 U/ml DNase (Sigma) for 45 min, and single-cell suspensions were prepared by passing through a 100-µm mesh filter (BD Biosciences). Cells were blocked with Fc blocker (anti-CD16/32—clone 2.4G2 clone) (BD Biosciences) for 15 min and incubated with CD11c beads and isolated as per manufacturer’ protocol. Cells were plated at 1 million per well in a 24-well plate, stimulated with CpG (2 h) and BKs (2 h) and harvested for RNA and analyzed by real-time RT-PCR for ISG expression.

### Flow Cytometry

Bone-marrow-derived dendritic cells were harvested from the wells, washed in cold PBS, incubated with Fc blocker (2.4G2 monoclonal antibody, BD Biosciences) for 10 min, and then stained for 30 min on ice with allophycocyanin-conjugated hamster anti-mouse CD11c, FITC conjugated mouse anti-mouse H2Kb (MHC Class I) (all antibodies purchased from BD Biosciences). Cells were fixed in 1% formaldehyde (eBioscience, San Diego, CA, USA) and analyzed on a FACS Canto II cytometer (BD Biosciences). FlowJo software (FlowJO LLC, Ashland, OR, USA) was used for data analysis.

### Gene Expression Analysis

Gene expression in BMDCs was analyzed by real-time RT-PCR using TaqMan probes. Briefly, RNA was extracted using Zymo quick kit (Zymo Research, Irvine, CA, USA) following the manufacturer’s protocols. Complementary DNA was synthesized using the cDNA archive kit (Life Technologies, Carlsbad, CA, USA). TaqMan primers and probes for both mouse and human genes (*Irf7*—Mm00516788_m1, *IRF7*—Hs01014809_g1; *Isg15*—Mm01705338_s1, *ISG15*-Hs01921425_s1, *Ifnβ*—Mm00439546_s1, and *Cxcl10*—Mm00445235, *CXCL10*-Hs00171042_m1) were purchased from Applied Biosystems (Foster City, CA, USA). Cyclophilin (Mouse—Mm02342430_g1) was used as the reference gene for normalization for mouse cells and *GAPDH* (Human—4310884E) was used for human cells. The primer sequences are available from the Applied Biosystems website. The cycle threshold (Ct) method of relative quantification of gene expression was used (ddCt), and the normalized Ct values (against cyclophilin or *GAPDH* for mouse and human cells, respectively) were calibrated against the control (untreated cells) in each experiment. Percent fold change was calculated by normalizing the values to the CpG, R848, or IFN-α treatments alone in each experiment. Mean and SEM were obtained from at least three independent experiments from independent cultures from different mice or different human donors, and are presented in the graphs.

### Western Blot Analysis

Western blotting was performed as described previously (25) using 25–50 µg of total proteins from DC cell lysates. In brief, protein samples were denatured by boiling for 10 min and loaded onto 4–20% Bis–Tris gels (Invitrogen, Carlsbad, CA, USA). After electrophoresis, proteins were transferred to nitrocellulose membranes. Membranes were blocked for 1 h with blocking buffer (2% non-fat milk in PBS), then incubated overnight at 4°C with the primary antibodies diluted in blocking buffer with 0.1% Tween 20. Rabbit polyclonal anti-STAT1 and STAT2, phospho-STAT1 (Tyr701) and phospho-STAT2 (Tyr689) (Millipore) were used as primary antibodies. Anti-actin Ab (Santa-Cruz Biotech) was used as a loading control. After incubating with primary antibodies, the membranes were washed with PBS containing 0.1% Tween 20 (PBST) three times. Membranes were then incubated for 1 h with IR Dye 800 goat anti-rabbit and IR Dye 680 goat anti-mouse (LI-COR Biosciences, Lincoln, NE, USA) diluted in blocking buffer plus 0.1% Tween 20. The blots were then washed three times with PBST and rinsed with PBS. Proteins were visualized by scanning the membrane on an Odyssey Infrared Imaging System (LI-COR Biosciences) in both 700 and 800 nm channels. ImageJ software (Image processing program developed at the National Institutes of Health) was used to calculate the band intensities.

### ELISA

We used ELISA kits (sandwich elisa) to measure the levels of mouse cytokines IL-6, IL12p70 (BD Biosciences, San Jose, CA, USA), and CXCL10 (R&D Systems, Minneapolis, MN, USA) in the supernatants of BMDC cultures stimulated for 24 h with TLR ligands, or medium alone, as per the manufacturer’s protocol.

### Statistical Analysis

Mean and SEM were calculated from data from three to seven independent experiments performed with independent bone marrow cultures or spleens obtained from individual mouse in each experiment or from at least three to five human donors performed independently. Prism software (GraphPad, San Diego, CA, USA) was used for statistical analysis: Two-tailed paired *t*-test for comparison between two groups and one-way ANOVA with Tukey’s *post hoc* test for multiple groups were used for statistic, as appropriate. *p*-values less than 0.05 were considered significant (marked in the figures as *p < 0.05, **p < 0.01, ***p < 0.001).

## Results

### BKs Suppress ISGs in Normal and Lupus-Prone Murine DCs *In Vitro* and in Human PBMCs

**Figure 1:**
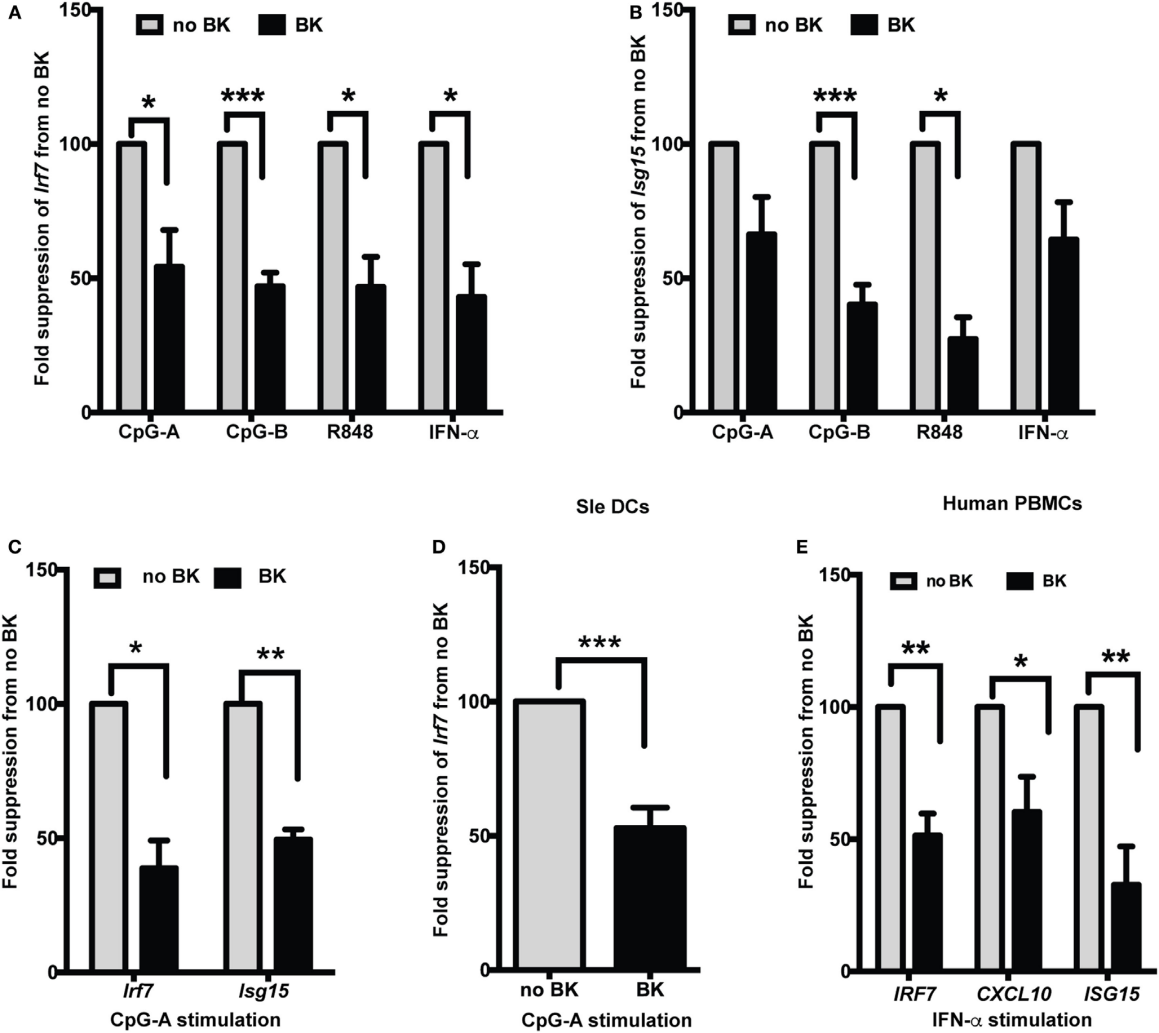
Bradykinins (BKs) suppress interferon-stimulated genes (ISGs) in mouse and human cells. **(A–C)** Murine bone-marrow-derived dendritic cells (BMDCs) grown in GMCSF containing medium were treated or not with CpG-A, CpG-B, R848, or recombinant IFN-α for 3 h, treated with BK for 4 h and then analyzed expression of *Irf7* **(A)** and *Isg15* **(B)** by real-time qPCR. **(C)** GMCSF dendritic cells (DCs) were stimulated overnight with CpG-A and with BK for a further 4 h and *Irf7* and *Isg15* responses were analyzed by qPCR. **(D)** Sle DCs grown in Flt3L containing medium were stimulated with CpG-A followed by BK and *Irf7* gene expression was analyzed. **(E)** Human PBMCs were stimulated with IFN-α for 3 h followed by BK for a further 3 h and then *IRF7, CXCL10*, and *ISG15* gene expression was analyzed. Percent fold change in expression was calculated by normalizing with no BK treatment for each stimulation in each experiment. Bar graphs represent the average (mean + SEM) of least three independent experiments, performed with independent BMDC culture from each mouse or PBMCs from each donor; *p < 0.05, **p < 0.01, ***p < 0.001.

Several studies have shown increased production of IFN-α in lupus, both in spontaneous lupus-prone mouse models (23, 32) and in patients (16, 33). We, and others, have also shown that stimulation *via* TLR7 and TLR9 is important in the induction of Type I IFNs in lupus (14, 19, 23). We, therefore, used ligands against TLR7 (R848) and TLR9 (CpG-A and -B) and also directly stimulated cells with recombinant IFN-α to test if the ISGs’ expression was modulated by BKs in normal and lupus-prone mouse DCs. We tested both a short-time (3 h) as well as long-time (20 h) stimulation with the TLRs and then added BKs without washing out the initial stimuli and analyzed the responses after 4 h. Using bone-marrow-derived DCs grown in GMCSF (GM-cDCs), a murine model of pro-inflammatory DCs, we tested three types of BKs, B1 and B2 receptor agonists and bradykinin peptide and we combined the results and presented in Figure 1. BKs significantly decreased the induction of *Irf7* upon short-time stimulation (total of 7 h) with CpG, R848, or IFN-α in B6 GM-cDCs (Figure 1A). The fold changes (mean ± SE) of *Irf7* from untreated control (that was normalized to 1) in GM-cDCs stimulated with TLR ligands without BK vs. with BK treatment were: CpG-A—14.9 ± 3.2 vs. 8.7 ± 5.2; CpG-B—53.4 ± 18.9 vs. 24.4 ± 8.7; R848—13.3 ± 2.4 vs. 6.0 ± 1.6; IFN-α—82.6 ± 48.8 vs. 37 ± 21.6. The ubiquitin-like *Isg15* gene is one of the predominant genes induced by Type I IFNs (IFNα/β) (34) that we have previously shown to be induced by CpG and R848 in B6 BMDCs (26). We found that BK significantly suppressed the gene expression of *Isg15* induced by CpG-B and R848 in GM-cDCs (Figure 1B). The fold changes (mean ± SE) of *Isg15* from untreated control (that was normalized to 1) in GM-cDCs stimulated with TLR ligands without BK vs. with BK treatment were: CpG-A—36.8 ± 6 vs. 26.1 ± 2.9; CpG-B—53.1 ± 23.4 vs. 30.5 ± 10.1; R848—26.8 ± 3.3 vs. 13.0 ± 4.9; IFN-α—179.9 ± 124.8 vs. 93.4 ± 50.1. To determine further whether BK can modulate a sustained IFN response, we stimulated B6 GM-cDCs with CpG-A for 20 h and then treated with BK for 4 h and analyzed *Irf7* (mean ± SE—no BK vs. BK—173.8 ± 47.3 vs. 89.5 ± 26.8) and ISG15 (no BK vs. BK—108.3 ± 28.8 vs. 55.7 ± 18.4) gene expression. BK significantly suppressed the ongoing production of both *Irf7* and *Isg15* genes in B6 GM-cDCs (Figure 1C). These results suggest that BK can also strongly suppress the autocrine positive feedback loop that Type I IFNs generate on ISG production upon a sustained response (35). We observed that *Isg15* suppression reached statistical significance after 20 h stimulation with CpG-A (Figure 1C) that was not observed at the shorter time (3 h) (Figure 1B).

We tested the effects of BK in the DCs from Sle mice and found that a sustained *Irf7* gene expression was strongly suppressed by BK (mean ± SE—no BK vs. BK—35.9 ± 9.7 vs. 17.3 ± 5.0) (Figure 1D). This suggests that BK can also suppress the IFN response ongoing in Sle DCs, which we have previously shown to be amplified compared to the IFN response of B6 DCs upon CpG stimulation (23).

We further wanted to determine if this phenomenon could be reproduced in human cells. We isolated PBMCs from normal human donors and stimulated them with recombinant IFN-α for 3 h and then we added BKs for another 3 h and analyzed the gene expression of *IRF7, CXCL10*, and *ISG15*. The upregulation of all three genes were strongly suppressed by BK (mean ± SE—no BK vs. BK—*IRF7*—21.5 ± 6.4 vs. 10.2 ± 1.9; *CXCL10*—748 ± 290.7 vs. 332.8 ± 78.2; *ISG15*—239.8 ± 48.1 vs. 54 ± 19.8) (Figure 1E). None of the BKs tested induced any IFN response by themselves in the mouse or human cells (data not shown). The concentrations of BKs used in these experiments also did not affect the DC percentage or cause any toxicity to the cells in culture (data not shown). By performing titrations, we found that BKs showed the strongest inhibition of the IFN responses at 10 µM.

### BKs Inhibit Type I IFN-Induced MHC Class I Upregulation, and CXCL10 Production in Murine DCs *In Vitro*

**Figure 2:**
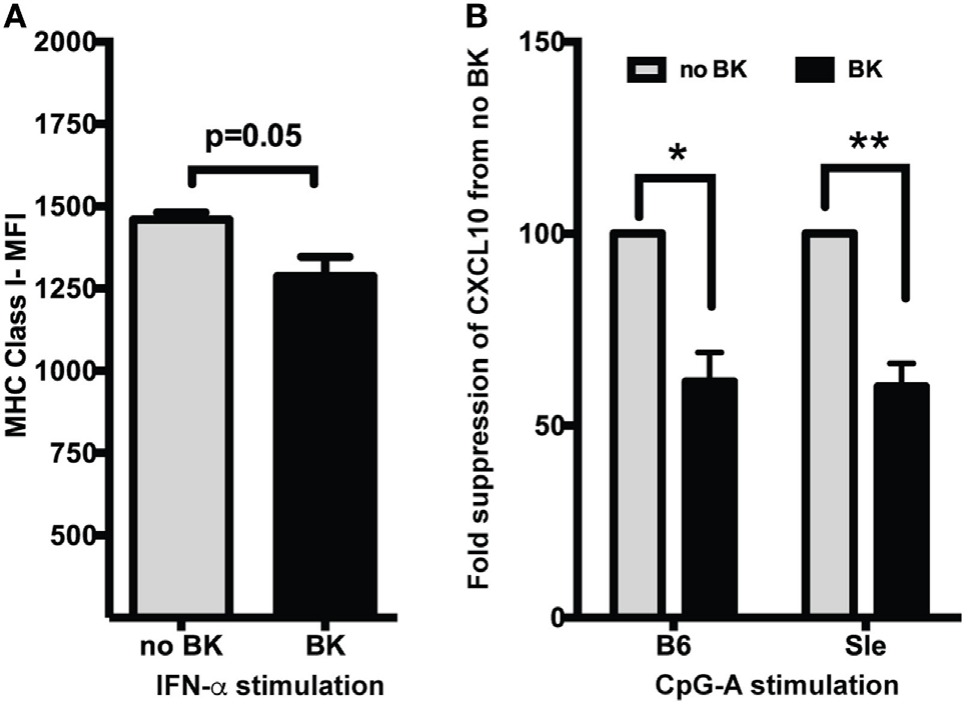
Bradykinins (BKs) suppress MHC Class I upregulation and CXCL10 production in murine dendritic cells (DCs). **(A)** Mouse bone-marrow-derived dendritic cells (BMDCs) were treated or not with recombinant IFN-α for 3 h and then stimulated with BK for 21 h. Cells were harvested and analyzed for MHC Class I expression by flow cytometry. Cells were also stained with CD11c and gated on CD11c DC populations. Data in the bar graphs represent averages (mean + SE) of median fluorescence intensity (MFI) of the markers analyzed from at least three independent cultures. **(B)** Supernantants from B6 and Sle BMDCs treated with CpG-A followed by BK for a total of 24 h were analyzed for CXCL10 production by ELISA. Percent change in CXCL10 production was calculated from no BK treatment for each experiment. Bar graphs are mean + SE of three independent experiments analyzed in duplicate in each experiment. *p < 0.05; **p < 0.01.

We have shown before that MHC Class I was strongly upregulated by Type I IFNs (25) and by TLR ligands *via* autocrine production of Type I IFNs (27). We have also shown that chemokine CXCL10 is very strongly upregulated in Sle DCs upon CpG stimulation (23) and that CXCL10 is a highly IFN-dependent cytokine (27). In this study, we tested if BKs can suppress the induction of these molecules. Treatment with IFN-α induced an increase in the expression of MHC Class I [untreated vs. IFN-α-median fluorescence intensity (MFI)—1178 ± 84 vs. 1460 ± 21] at 24 h in B6 GM-cDCs, as analyzed by flow cytometry. BK inhibited this MHC Class I upregulation (Figure 2A), with a small but consistent decrease (differences in MFI reached *p* = 0.05). We also measured the production of CXCL10 in supernatants from mouse DC cultures generated in Flt3-L-conditioned medium [which are considered a model of sentinel DCs and a mixed population of cDCs and pDCs (36)], by ELISA after 24 h stimulation with CpG-A. BK strongly suppressed the production of CXCL10 upon CpG stimulation in both in B6 and Sle DCs (Figure 2B; mean ± SE—CXCL10 picograms/milliliter—no BK vs. BK—B6—4,383 ± 80 vs. 2,689 + 1; Sle—2,322 ± 1,354 vs. 1,331 ± 736). We also tested the BK effects on CXCL10 induced by R848, but interestingly BK did not suppress this response (data not shown). These results suggest that BK inhibit ISG responses both at RNA transcription and protein translation levels.

### BKs Strongly Suppress Pro-inflammatory Cytokine Production in Murine DCs

**Figure 3:**
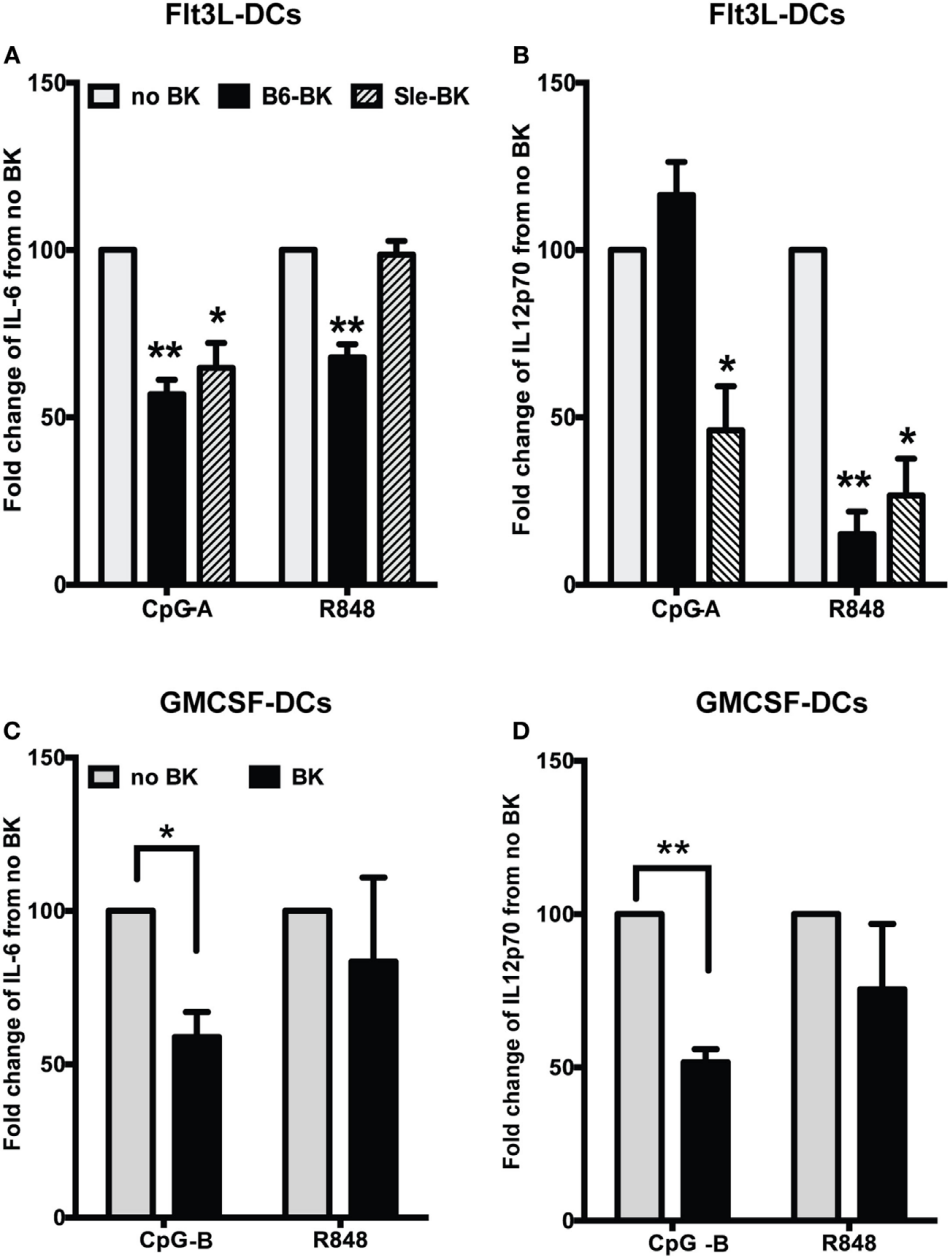
Bradykinins (BKs) suppress pro-inflammatory cytokine production in murine bone-marrow-derived dendritic cells (BMDCs). **(A,B)** Flt3L grown BMDCs from B6 or Sle mice were stimulated with CpG-A or R848 overnight and then stimulated with BK. Cell supernatants were collected at 24 h of total stimulation to analyze the cytokines, IL-6 **(A)** and IL12p70 **(B)** by ELISA. **(C,D)** GMCSF-grown BMDCs from B6 mice were stimulated with CpG-B or R848 overnight and then stimulated with BK and cell supernatants were collected at 24 h of total stimulation to analyze the cytokines, IL-6 **(C)** and IL12p70 **(D)** by ELISA. Percent change in cytokine production was calculated from no BK treatment for each experiment. Bar graphs are mean + SE of at three independent experiments (*p < 0.05; **p < 0.01).

We next analyzed the effects of BK on pro-inflammatory cytokines IL-6 and IL12p70, in the supernatants of B6 and Sle BMDCs cultured in Flt3L medium (Figure 3A). We observed that BK significantly inhibited the production of IL-6 in B6 (*p* < 0.01) and Sle DCs (*p* < 0.05) upon CpG-A stimulation (Figure 3A; Figure S1A in Supplementary Material). There was a significant reduction of IL-6 upon R848 stimulation in the B6 DCs but not in the Sle DCs (Figure 3A; Figure S1B in Supplementary Material). Interestingly, IL12p70 was not downregulated by BK upon CpG-A stimulation in the B6 DCs, but was strongly suppressed in the Sle DCs (Figure 3B; Figure S1C in Supplementary Material). However, IL12p70 induced by R848 was strongly suppressed by BK both in the B6 and Sle BMDCs (B6, *p* < 0.01; Sle, *p* < 0.05) (Figure 3B; Figure S1D in Supplementary Material). We also tested the effect of BK in the GM-cDCs and found that CpG-B induced IL-6 and IL12p70 were significantly reduced by BK (Figures 3C,D; Figure S2 in Supplementary Material), while the same cytokines were unaffected by BK upon R848 stimulation (Figures 3C,D; Figure S2 in Supplementary Material). Untreated B6 or Sle DCs did not make detectable cytokines. In total, the results indicate that BK can suppress the upregulation of ISGs in both GMCSF or Flt3L -DC cultures (Figures 1A–D), but the effects on the cytokines upregulated by specific stimuli (CpG vs. R848) were very different in the different cultures, suggesting that there may be some post-translational effects that vary in the mixed Flt3L cultures vs. the GMCSF cDC cultures. Moreover, we observed a more consistent suppressive effect of BK on the cytokines induced by CpG (A and B) but not with R848, suggesting different susceptibility to BK of the distinct signaling pathways downstream of TLR9 and TLR7 (Figures 3C,D and 2B).

### Effects of BKs on the Phosphorylation of Signal Transducers and Activator of Transcription (STATs) upon IFN-α Stimulation in Murine DCs

**Figure 4:**
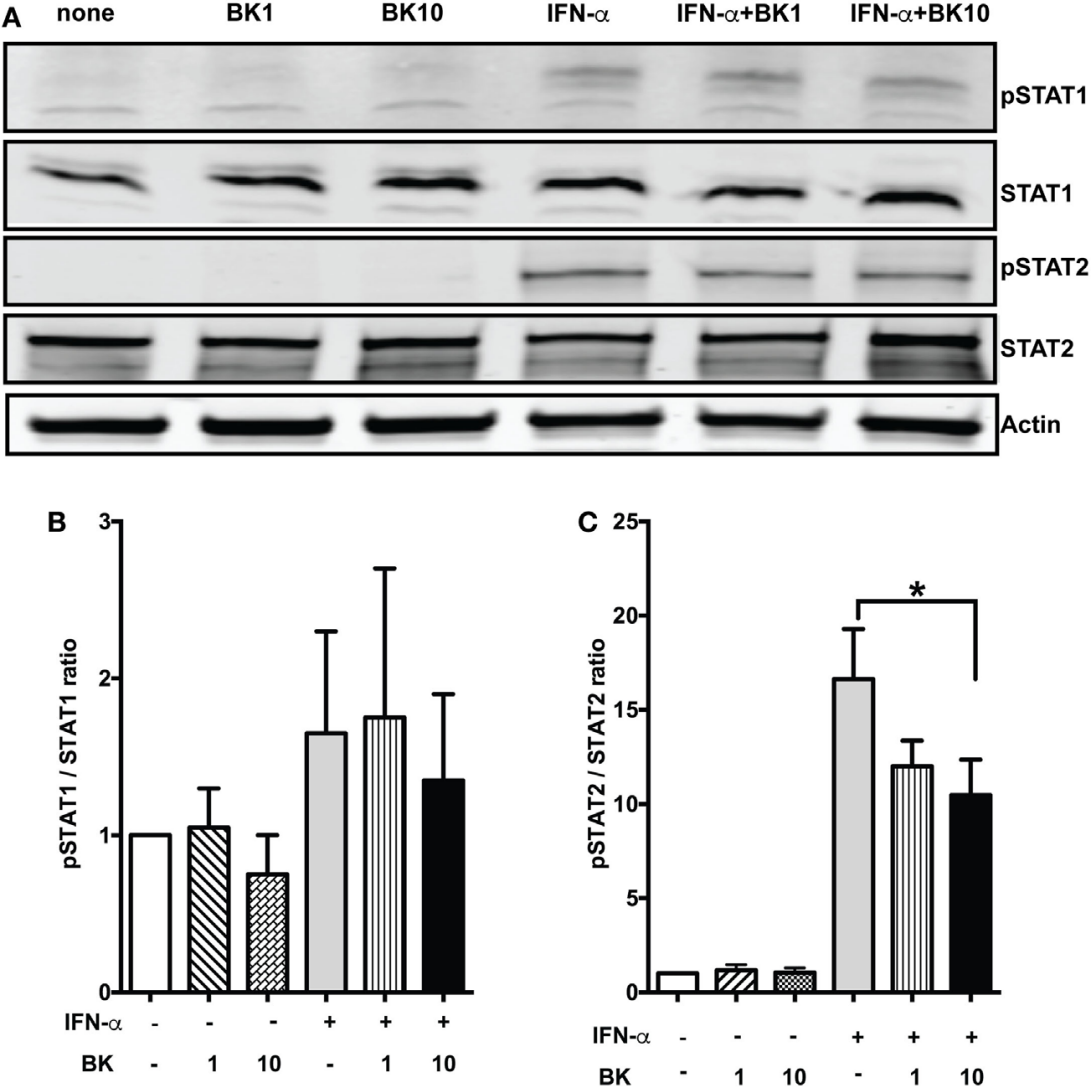
Bradykinins (BKs) suppress STAT2 phosphorylation. **(A)** Phosphorylation (p) and expression of STAT1 and STAT2 in bone-marrow-derived dendritic cells (BMDCs) stimulated with IFN-α for 15 min followed by BK for 30 min was analyzed by western blot. Actin was used as loading control. **(B,C)** Bars represent the ratios of intensities of each pSTAT and corresponding signal transducer and activator of transcription band, normalized to the respective loading controls. A representative blot of three independent experiments conducted with three independent BMDC cultures is shown. The differences in pSTAT2/STAT2 reached statistical significance; IFN-α—no BK vs. IFN-α-BK 10—*p < 0.05.

Classical Type I IFN signaling takes place *via* IFNAR1 and IFNAR2 receptors that activate the Janus kinase–STAT pathway, leading to transcription of ISGs (37). To determine the effects of BK on the signaling pathway downstream of IFNARs, we chose to analyze the effects on the phosphorylation of STAT1 and STAT2 molecules. We found that IFN- α-induced phosphorylation of STAT2 was suppressed by BK, while there was moderate effect on STAT1, as analyzed by western blotting. BK alone did not induce any phosphorylation of STAT1 or STAT2 (Figure 4). These results indicate that BK might suppress the activation of key molecules that mediate the IFN signaling. Since STAT1 is downstream of the receptors of other cytokines like IFN-γ, while STAT2 molecule is specifically downstream of Type I IFN signaling (37), our results suggest that BK may specifically regulate Type I IFNs.

### BKs Suppress ISGs in Normal and Lupus-Prone Murine Splenocytes *In Vivo* and DCs of Normal Mouse Spleen *Ex Vivo*

**Figure 5:**
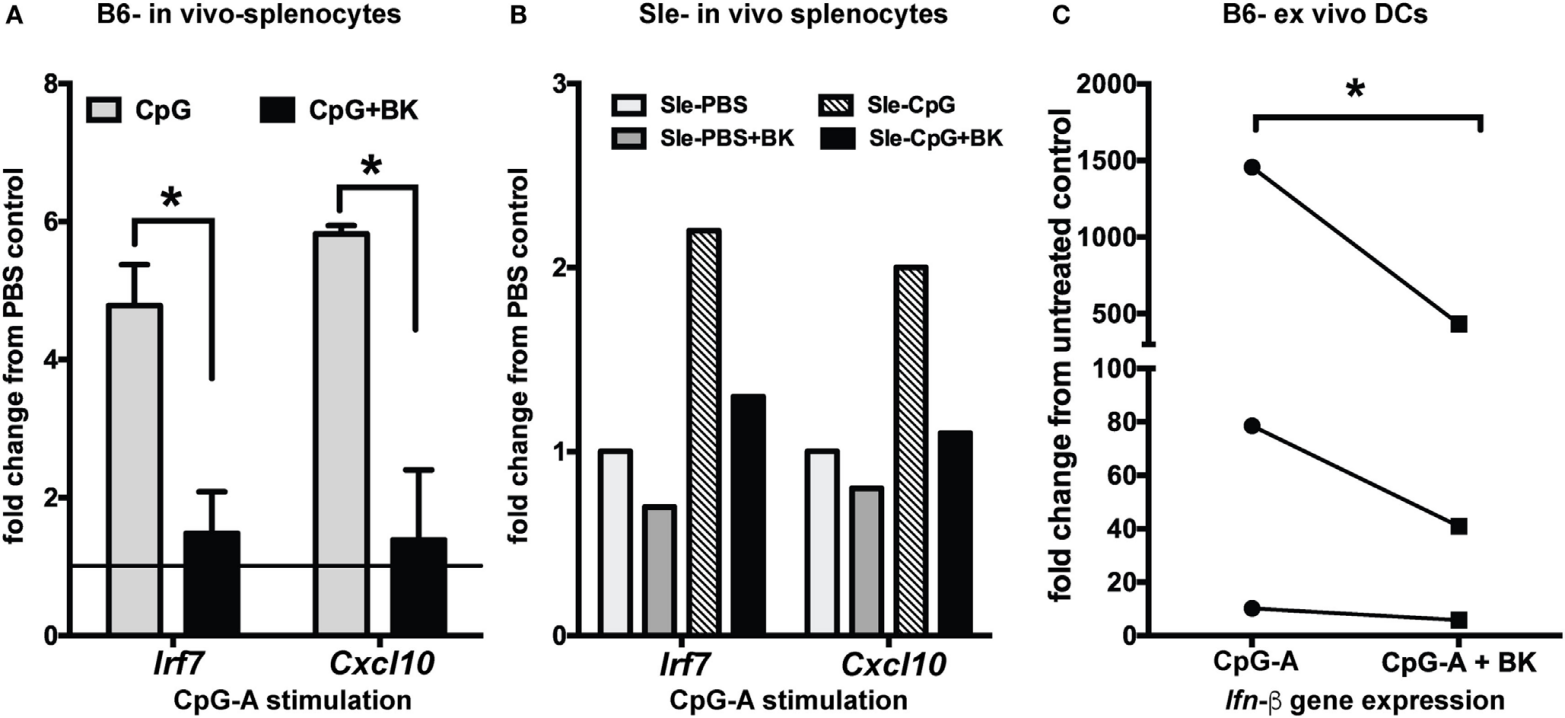
Bradykinins (BKs) suppress interferon-stimulated genes in splenocytes *in vivo* and in *ex vivo* mouse dendritic cells (DCs). **(A)** B6 and Sle mice were injected with CpG-A intraperitoneally for overnight stimulation, BK was administered retro-orbitally for 2 h and expression of the IFN-inducible genes was analyzed from total splenocytes by real-time qPCR. All of the conditions were calibrated against the untreated control in each experiment. Graphs are averages of three independent B6 mice sets **(A)** and one representative Sle set **(B)**. **(C)** DCs from B6 mouse spleen were sorted by Miltenyi magnetic beads, plated in 24-well plates, stimulated with CpG-A (2 h) followed by BK (3 h) and analyzed for *Ifn-β* expression. Line graphs are fold change in gene expression normalized to untreated control in each experiment; *p < 0.05.

To determine the effects of BK *in vivo*, we analyzed the effects of CpG-induced IFN responses both in B6 and Sle mice. We injected CpG-A intraperitoneally in mice and, after overnight stimulation, we administered BK retro-orbitally. BK is a short-lived peptide (38) and, therefore, the *in vivo* stimulation was performed very quickly and 2 h after BK injection we collected the spleen and isolated RNA from total splenocytes to analyze the expression of the IFN-inducible genes by real-time qPCR. We found a strong induction of *Irf7* and *Cxcl10* upon CpG stimulation both in the B6 and Sle splenocytes, that was significantly downregulated upon BK administration (*p* < 0.05) (Figures 5A,B). We also observed that the constitutive expression *Irf7* and *Cxcl10* expression was also slightly downregulated in the Sle splenocytes (Figure 5B). The constitutive expression of ISGs in the Sle splenocytes was greater than twofold as compared to the B6 splenocytes (data not shown). To further determine the effects of BK on *in vivo* differentiated DCs, CD11c positive DCs from spleens of B6 mice were sorted and cultured *ex vivo*. We stimulated the splenic DCs with CpG for 2 h and then with BK for 3 h. We found that BK strongly suppressed the induction of *Ifn-β* (*p* < 0.05; Figure 5C) indicating that BK is a negative regulator of the IFN response in immune cells in general, and in cDCs in particular, *in vitro* and *in vivo*.

### klks and Captopril (ACEi) Suppress IFN-Induced ISGs in Murine DCs and Human PBMCs

**Figure 6:**
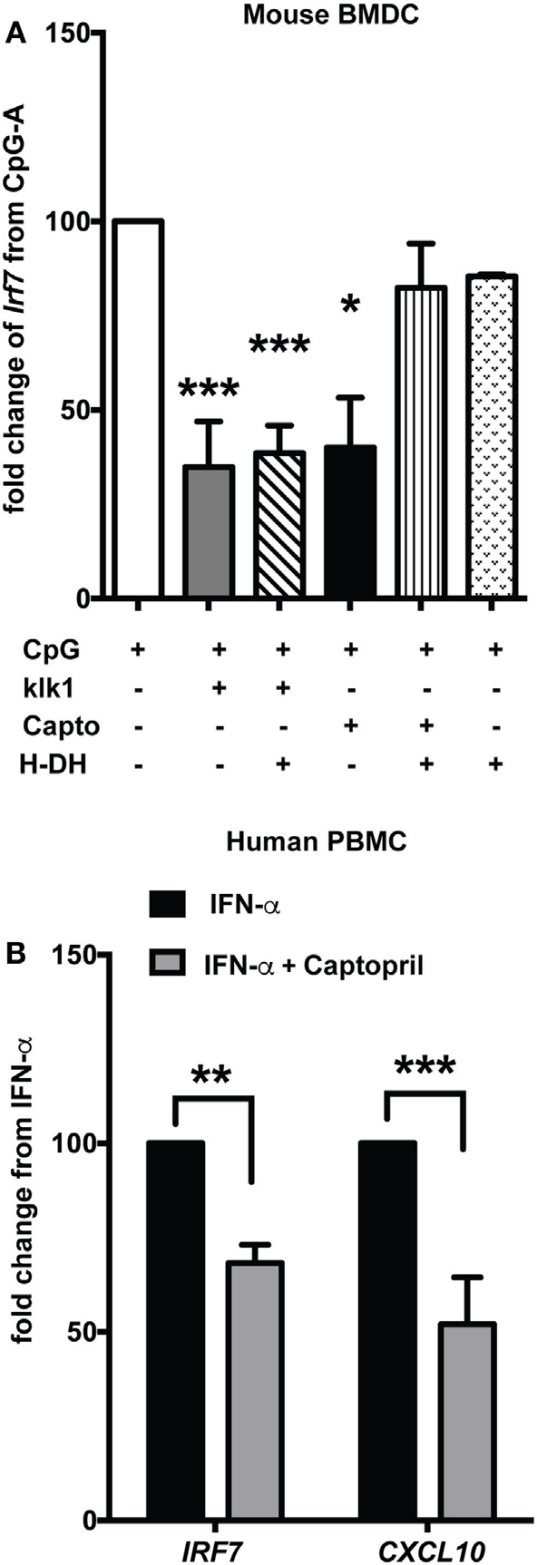
klk1 and captopril also suppress interferon-stimulated genes. **(A)** Murine bone-marrow-derived dendritic cells (BMDCs) were stimulated with CpG-A for 2.5 h treated with antagonists for 30 min to block both B1 (DH—1 µg/ml) and B2 (H—10 µM) receptors and then stimulated with klk1 (1 µg/ml) or captopril (20 µM) for a further 4 h. *Irf7* gene expression was analyzed by qPCR. Percent fold change was calculated by normalizing to CpG-A stimulation alone. **(B)** Human PBMCs were first treated with IFN-α for 3 h, 20 µM captopril was added and harvested after further 3 h and *IRF7* and *CXCL10* gene expression was analyzed by qPCR. Percent fold change was calculated by normalizing with IFN-α stimulation alone. Panels **(A,B)** represent average (mean + SEM) of at least three independent experiments, performed with independent BMDC cultures from each mouse or PBMCs from each donor; *p < 0.05, **p < 0.01, ***p < 0.001.

Earlier reports have shown that antibody-induced GN in lupus-prone mice is ameliorated by exogenous administration of klk1, one of the molecules of the KKS (21). In order to determine if klks can affect Type I IFN responses *in vitro*, we induced an IFN response with CpG-A and then added recombinant klk1 (1 µg/ml) and analyzed *Irf7* gene expression. Klk1 significantly suppressed CpG-A-induced *Irf7* gene expression in mouse DCs (*p* < 0.001) (Figure 6A). To further test if klk1 acted *via* BK signaling to bring about this suppression, we blocked both B2 and B1 receptors with their inhibitors HOE140 (H) (10 µM) and des-Arg^10^-HOE140 (DH) (1 µg/ml), respectively. Klk1 could suppress *Irf7* gene expression even after blocking the BK receptors (*p* < 0.001), suggesting that klk may use an alternate and distinct pathway, specific of klks, possibly *via* protease-activated receptors (PARs) to bring about this suppression. To further test other important candidates of the KKS system, we focused on ACE, because captopril, an ACE inhibitor is a widely prescribed drug for lupus patients with kidney disease (10, 39) and we anticipated captopril to have other immunoregulatory effects (40) other than regulating blood pressure. We measured the effects of captopril (20 µM) on ISGs in mouse DCs and found that it can efficiently suppress *Irf7* gene expression (*p* < 0.05) (Figure 6A). Blocking of the BK receptors and then giving captopril reverted the suppressive effect, suggesting that captopril may primarily affect the availability of BK to signal *via* BK receptors on the cDCs to bring about downregulation of IFN-induced responses. Blocking the B1 and B2 receptors with the respective antagonists at the indicated concentrations did block the effects of further stimulation with BK, as analyzed by *Irf7* gene expression (data not shown). A strong suppression of *IRF7* (*p* < 0.01) and *CXCL10* (*p* < 0.001), gene expression was also observed in human PBMCs stimulated with IFN-α and then treated with captopril (20 µM) (Figure 6B).

### BKs Suppress IFN Responses *via* PGE2

**Figure 7:**
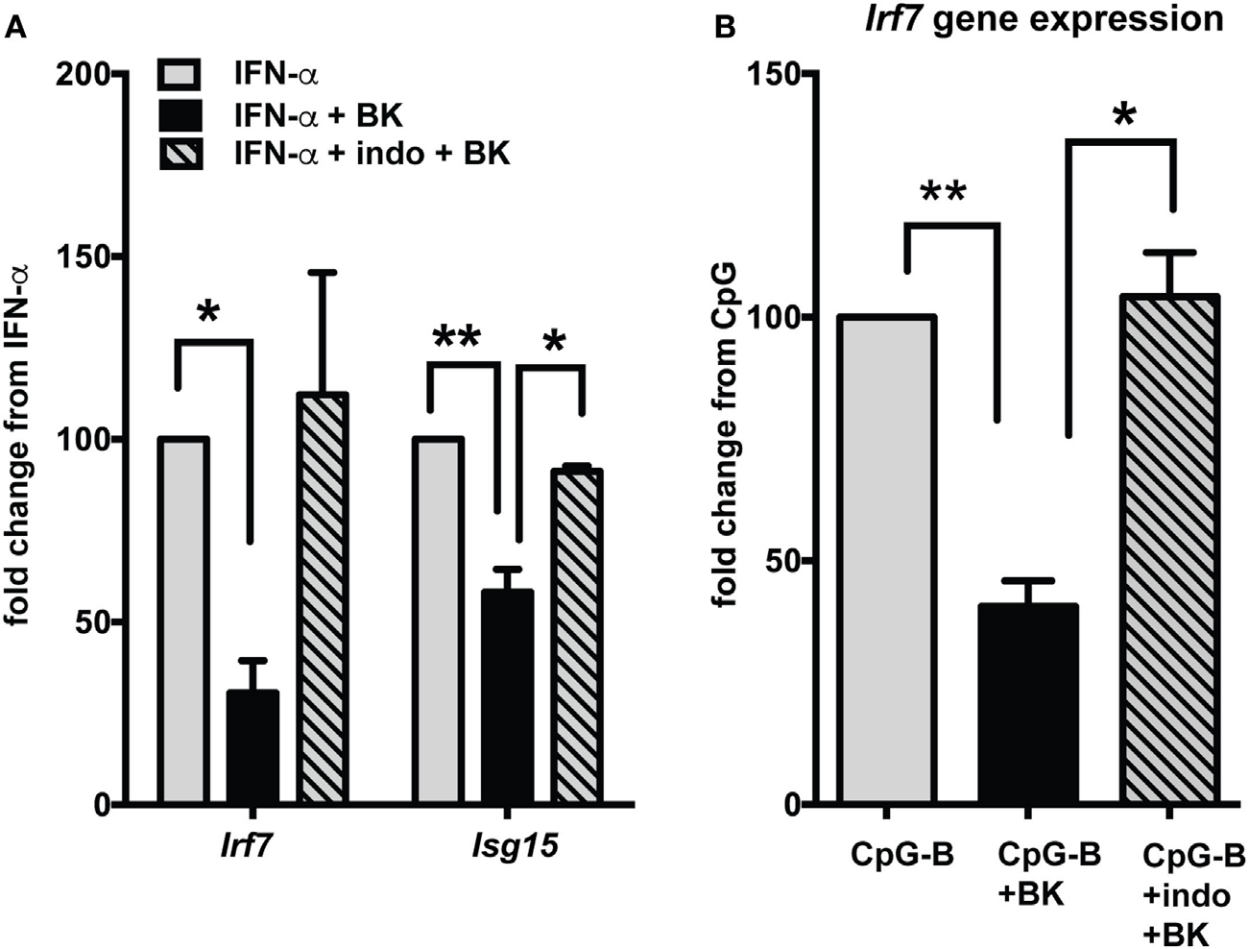
Bradykinin (BK) suppression of interferon-stimulated genes (ISGs) is mediated *via* PGE2. Murine bone-marrow-derived dendritic cells were stimulated with IFN-α **(A)** or CpG-B **(B)** for 2.5 h then 1 µg/ml indomethacin (indo) (COX1 and -2 inhibitor) was added and incubated for 30 min followed by stimulation with BK for a further 4 h. Cells were harvested and ISGs were analyzed by qPCR. Percent fold change was calculated by normalizing with IFN-α stimulation alone **(A)** or CpG-B stimulation alone **(B)**; *p < 0.05; **p < 0.01.

Production of prostaglandins *via* BK receptor signaling has been documented in several studies (41, 42). A recent study indicates that prostaglandins, especially PGE2 can suppress IFN responses in pDCs (43). To determine if the BK suppression of IFN responses is mediated *via* PGE2, we used indo to inhibit PGE2 production and analyzed ISG expression (Figure 7). The BK-induced suppression of ISGs, both with direct IFN stimulation (Figure 7A) or upon TLR (CpG-B) stimulation (Figure 7B), was significantly reverted with indo treatment, suggesting that BKs, may bring about suppression of IFN responses, at least in part, *via* prostaglandins.

## Discussion

Studies in the past decade have discovered the role of the KKS in regulating immune responses (5, 44) in several models of autoimmunity (45) including lupus (12, 46) and cancer (47). Type I IFNs have been investigated both for their pathogenic role in diseases such as lupus (48, 49) and for its therapeutic role as in MS (50) or in some forms of cancer and chronic viral infections (51). Besides the classic pathways of Type I IFN production *via* the TLRs, many cytosolic nucleic acid sensors have also been shown to induce IFN production (52) adding to the complexity of the regulatory mechanisms of the IFN system (53). Although the role of KKS has been studied in some immunological pathways (44, 54–57), the effect of this system in regulating IFN responses has never been addressed.

In order to determine the effects of KKS in modulating Type I IFN responses, we tested the BKs which are central in the KKS system (1, 21, 58), tissue klk1 which has been shown to ameliorate GN in antibody-mediated kidney disease (12) and captopril, a widely prescribed ACEi (10, 59). We clearly demonstrated that all these molecules suppressed Type I IFN responses induced by the classical TLR pathway (using TLR7 and -9 agonists) or directly by recombinant IFN-α. BKs suppressed important ISGs that we have previously shown to be induced upon TLR7 and -9 stimulation in lupus dendritic cells (DCs) (23), especially the IRF7, a key regulator of Type I IFN responses (60). BKs efficiently suppressed both short and sustained IFN responses in DCs of normal and lupus-prone Sle mice grown *in vitro*. We have previously reported induction of MHC Class I as an IFN-specific response (25). The observation that BKs can suppress the upregulation of MHC Class I may have important implications for the innate IFNs response during viral infections (61). To study the immunomodulatory effects of BK, we used two *in vitro* models of DCs, the GM-cDCs and the FLt3L-DCs and found similar results, indicating that BK can regulate different subsets of DCs. Moreover, the induction of IFN responses in splenocytes *in vivo*, as well as in mature splenic DCs isolated and stimulated *ex vivo*, were also strongly suppressed by BK. Altogether, these results suggest that BKs are important negative regulators of the Type I IFN response in immune cells. The BK suppressive effect of IFN responses is further validated by our demonstration of this phenomenon in human PBMCs. The results suggest that different cell types in human blood show decreased responses upon BK stimulation, similar to mouse DCs studied *in vitro*.

Bradykinins also suppressed the IFN-induced phosphorylation of STAT2 molecules, a key step for further signaling, translocation to the nucleus and induction of IFN responsive genes (37, 62). However, suppression of STAT1 phosphorylation was only marginal. Since STAT1 is downstream of many cytokine receptors such as IFN-γ, while STAT2 is specifically downstream of Type I IFN receptor (62), our results suggest that BK may specifically regulate Type I IFNs. Taken together, these results strongly indicate that signaling *via* BK has an important role in regulating IFN-induced responses.

We have shown before that CXCL10 is a strongly IFN-dependent cytokine (27). Serum CXCL10 has been shown to be increased in patients with various autoimmune diseases including rheumatoid arthritis, systemic lupus, and Sjogren’s syndrome (63). Urine CXCL10 has been significantly correlated with renal activity in lupus kidney disease (64), and CXCL10 has also been shown to be very high in the cerebrospinal fluid of patients with central nervous system lupus (65). In this study, we observed a strong suppressive effect of BK on CXCL10 both at gene (Figure 1E) and protein levels (Figure 2B), suggesting that drugs that increase BK, such as ACEis (Figure 6B), may downregulate this cytokine in lupus disease and thereby bring about beneficial effects.

Bradykinins have been shown to be pro-inflammatory and induce IL12 production in DCs (28) and IL-6 production in airway smooth muscle cells (66). The BKs that we tested in our DC cultures did not induce production of IL12p70, IL-6, or CXCL10 (data not shown). The IL12p70 production reported by Aliberti et al. (28) was observed in splenic DCs and their responses could be different from *in vitro* cultured BMDCs. Nevertheless, we found that BK inhibited the production of TLR-induced proinflammatroy cytokines, a result consistent with the inhibition of the IFN responses in DCs shown in this study.

We also found that tissue klk1 decreased the IFN response. klks are serine proteases that cleave low molecular-weight kininogen into BKs that exert their biological functions *via* triggering the kinin receptors, B1R and B2R (1). Liu et al. have indicated that *klks* are protective disease-associated genes in immune complex–induced nephritis and lupus nephritis in mice and in SLE patients (21). Our results are consistent with these findings and suggest that some of the protective effects of klks in lupus could be due to dampening IFN responses. Recent studies suggest that klk1 may also activate protease-activated receptors (PARs) in inflammatory diseases (67). PARs are a subfamily of G protein-coupled receptors that participate in cell signaling *via* cleavage or activation by klk1. Our results suggest that the regulation of IFN responses by klk is independent from the known kk regulation of BK and their receptors; other pathways such as PARs may be involved in IFN regulation and warrants further investigation.

Angiotensin-converting enzyme inhibitors (ACEi), which increase kinin availability *in vivo* (2), have been found to be beneficial in ameliorating kidney disease in mouse models (39, 68) as well as lupus patients (10, 22). Captopril is one of the most popularly prescribed drugs to control blood pressure (69, 70). ACEis have also been shown to be beneficial in other autoimmune diseases, such as rheumatoid arthritis (71), and prevention of complications in insulin-dependent diabetes mellitus (72). The suppressive effects of captopril on the IFN-induced ISGs in PBMC makes it even more important to further investigate the pathways that lead to the beneficial effects of this drug, not only in lupus but in other diseases as well. The rescue from the suppression of ISGs by captopril by blocking of the kinin receptors clearly indicates that captopril suppresses IFN response by increasing the BKs. It will be interesting to study the effects of other ACEis in regulating IFN responses.

To determine the molecular mechanism the mediate the regulation of the interferon response by BK, we considered some key molecules, including PGE2 (37). PGE2 is a major lipid mediator that is released at the sites of inflammatory and it has been shown to possess pro-inflammatory (73) and anti-inflammatory (74) properties depending on the cell type, the stimulation and the experimental models of inflammatory/autoimmune diseases used. In particular, PGE2 regulated inflammation in several cell types: PGE2 has been shown to be an important inhibitory modulator of airway inflammation (75); PGE2 reduced IL12 pro-inflammatory cytokine production in DCs (76) and also inhibited IL-27 production (76, 77). The role of PGE2 in IFN regulation has been reported recently (39). Production of PGE2 by monocytes has been shown to be one of the mechanisms by which IgG immunoglobulin attenuates IFN production in pDCs (39). Our results of the rescue of the inhibitory function of BK on the IFN response by indo, that inhibits the production of PGE2, suggest that this novel pathway of Type I IFN regulation by the KKS presented in this study is mediated *via* production of PGE2.

Type I IFN signaling refuels an autocrine positive feedback loop and amplifies its response (78). The system is controlled at several negative check-points such as the SOCS molecules (suppressors of cytokine signaling) (79). The dysregulation in these pathways may increase the production of IFN and cause more amplified responses in lupus (79). Our results indicate that BKs can suppress the ongoing IFN response efficiently, a pathway that is very promising to attenuate the IFN responses in this disease. The results that molecules of the KKS and molecules that induce an increase in BKs, such as the FDA approved ACEis, can suppress an ongoing IFN response suggest that they can be therapeutic in a clinical situation of chronic immune response, as in lupus disease that is characterized by a consistent IFN signature. This phenomenon warrants further investigation to design better therapeutic strategies for IFN-mediated autoimmune diseases.

## Ethics Statement

Animals were maintained in the animal facility in accordance to the guidelines of the Institutional Animal Care and Use Committees of Temple University, which is an American Association for the Accreditation of Laboratory Animal Care-accredited facility. According to Temple University IRB, human samples that are procured in an already de-identified state is not considered “human subjects research,” The samples received from American Red Cross, used in this study were de-identified samples and no information about the donors is available to the investigators.

## Author Contributions

US conceived the idea, designed experiments, and wrote the manuscript. AS, ML, NF, VR, MD, and US performed experiments and analyzed the data. All authors contributed to interpreting the data and writing of the manuscript. All authors have reviewed and approved the manuscript, and agreed with its submission.

## Conflict of Interest Statement

The authors declare that the research was conducted in the absence of any commercial or financial relationships that could be construed as a potential conflict of interest.
