# Supplementary figures and images for: Kallikrein–Kinin System Suppresses Type I Interferon Responses: A Novel Pathway of Interferon Regulation
Source: Front Immunol. 2018 Feb 2;9:156. doi: 10.3389/fimmu.2018.00156 (PMC5801412; doi:10.3389/fimmu.2018.00156)

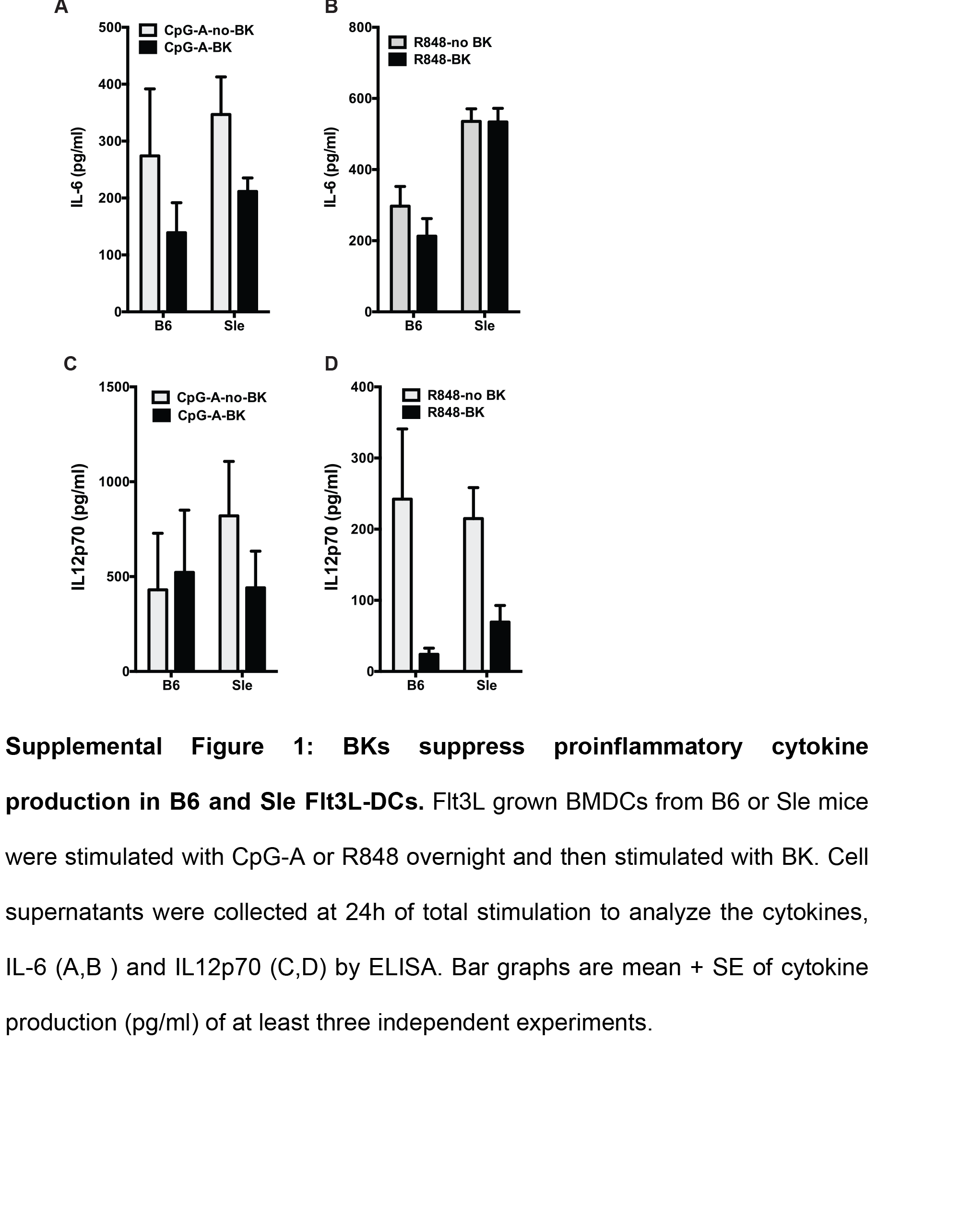

Supplement: Supplementary file 1 [file Image_1.tif]

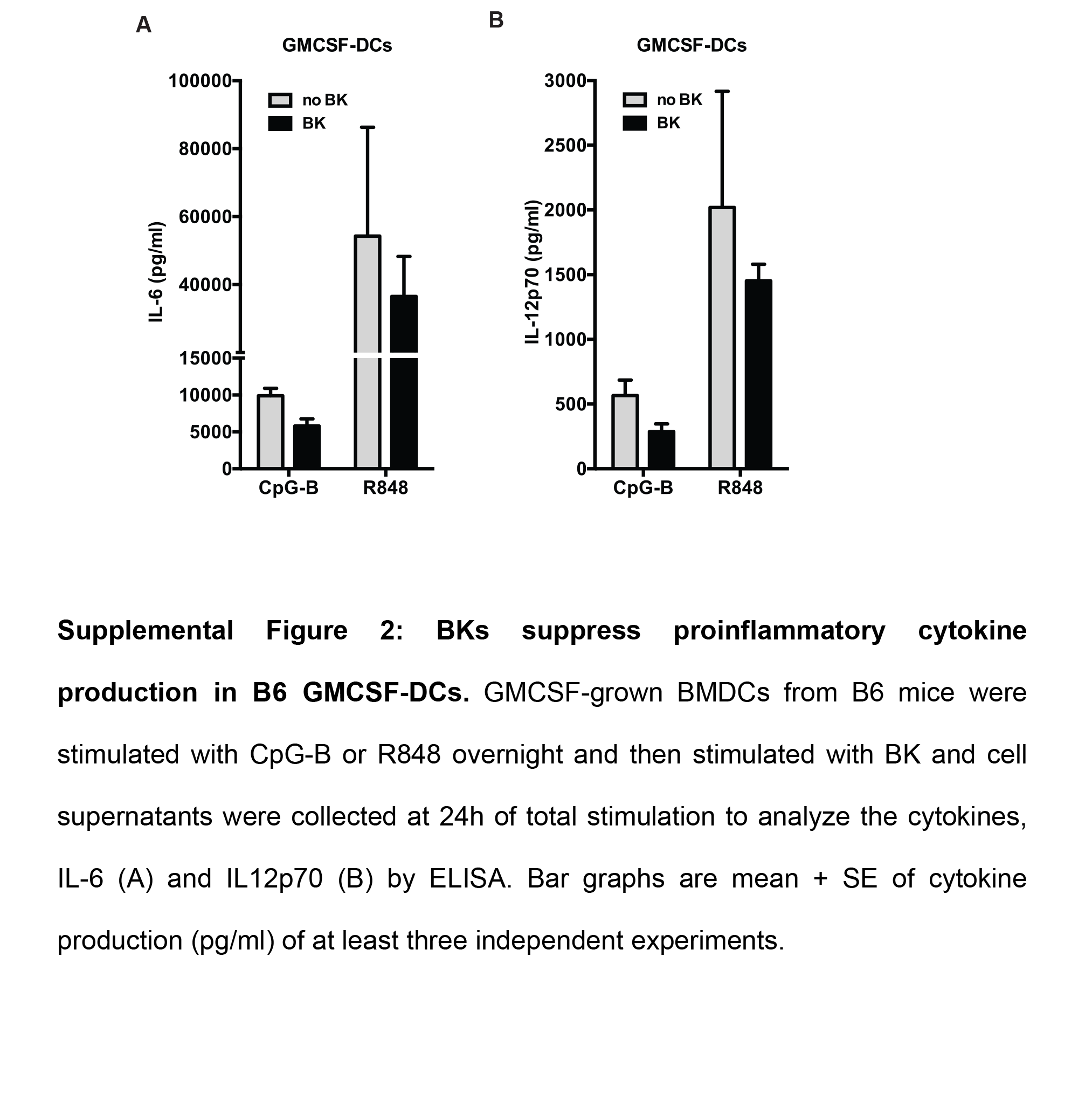

Supplement: Supplementary file 2 [file Image_2.tif]
